# Isolated CNS relapse of medullary aggressive high-grade B-cell lymphoma on ^18^F-FDG-PET/CT

**DOI:** 10.1186/s41824-022-00130-9

**Published:** 2022-05-03

**Authors:** Gerard Lambe, Simon Doran, Ruth Clifford, Afshin Nasoodi

**Affiliations:** 1 Radiology Department, Mater Misericordiae University Hospital, Eccles Street,, Dublin 7, Ireland; 2 Radiology Department, St. James’s Hospital, James’s Street, Dublin 8, Ireland; 3 Haematology Department, University Hospital Limerick, St Nessan’s Road, Dooradoyle, County Limerick, Ireland

**Keywords:** Positron emission tomography, Secondary CNS lymphoma, Spinal cord

## Abstract

This is a case of high-risk, aggressive, high-grade medullary B-cell lymphoma presenting with new onset of neurological dysfunction following initial complete response to the standard chemoimmunotherapy. A whole-body re-staging PET using fluorodeoxyglucose (^18^F-FDG) integrated with computed tomography (^18^FDG-PET/CT) performed with clinical suspicion of arachnoiditis, eloquently demonstrated unequivocal multifocal FDG uptake by the spinal cord without evidence of systemic recurrence, leading to a clinical diagnosis of secondary CNS lymphoma, which is a rare complication of DLBCL with ominous prognosis. Four cycles of Modified-MATRIX protocol resulted in a halt in fulminant course of the disease and the patient experienced slight reversal of the neurological deficits, although not deemed clinically fit for a repeat ^18^FDG-PET/CT due to his poor general well-being. Repeat MRI was suggestive of partial recovery, however. The clinical stability was proven short-lived, and the patient experienced progressive lower limb weakness only 3 weeks after discharge following his last cycle of treatment. Isolated CNS relapse of lymphoma is a rare occurrence in the literature. The CNS recurrence is more often leptomeningeal or confined to the brain parenchyma rather than the spinal cord. The role of ^18^FDG-PET/CT in the diagnostic algorithm of secondary CNS lymphoma is unclear and its significance in risk stratification and assessing the response to treatment has not been evaluated. This case report illustrates the imaging findings of a more unusual form of the disease with multifocal intramedullary involvement of the spinal cord, and highlights imaging features of this rare condition with ^18^FDG-PET/CT and MRI to support decision making in good clinical practice.

## Main text

A 66-year-old Caucasian man was diagnosed with aggressive medullary high-grade Diffuse large B-cell lymphoma (DLBCL) with a BCL2 amplification based on marrow histology. He was treated with six cycles of conventional therapy with Rituximab, Cyclophosphamide, Doxorubicin, Vincristine and Prednisolone (R-CHOP). Intrathecal Methotrexate was commenced as the patient was deemed at high-risk of CNS relapse based on his high CNS-IPI (Central Nervous System International Prognostic Index) score, but the latter was withheld after two cycles due to intolerance. This resulted in a Deauville response score of 1 based on an end of treatment PET using fluorodeoxyglucose (^18^F-FDG) integrated with computed tomography (^18^FDG-PET/CT) study.

The patient presented only two weeks post completion of treatment with progressive right-sided visual loss followed by left lower limb weakness a week later. MRI (magnetic resonance imaging) of the brain and spine revealed multiple enhancing lesions throughout the spinal cord and suggested bilateral optic nerve involvement. High dose methylprednisolone was administered intravenously for three days during the investigation phase with the working diagnosis of possible arachnoiditis as a complication of chemotherapy and a re-staging whole body ^18^F-FDG-PET/CT scan was arranged to exclude systemic recurrence.

**Fig. 1:**
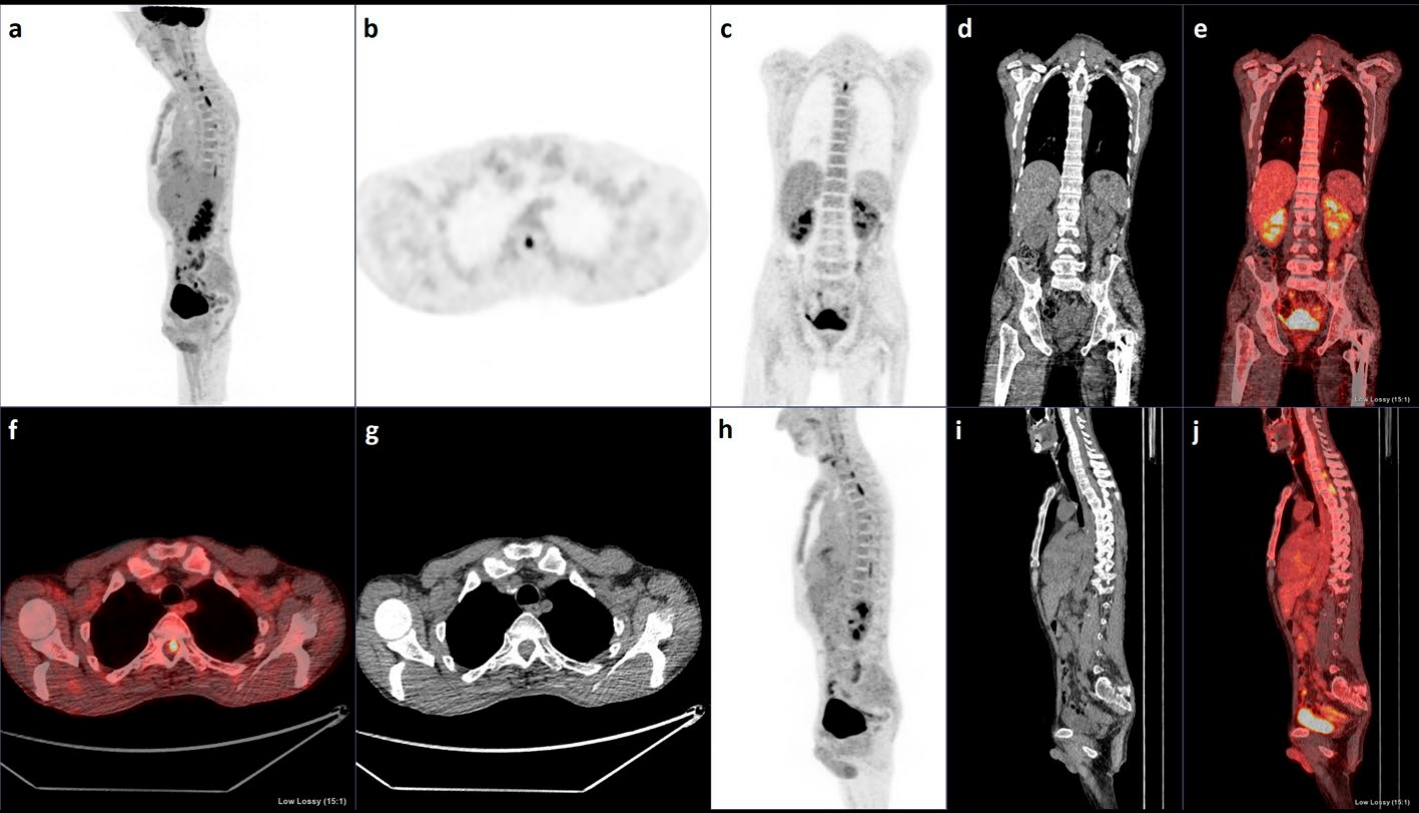
Sagittal MIP image (**a**) from whole-body ^18^F-FDG-PET/CT demonstrates intense multifocal segmental uptake in the cervical and thoracic spine (SUVmax 9.6). Axial (**b, f, g**), coronal (**c, d, e**) and sagittal (**h, i, j**) images confirm the intramedullary location of the lesions

As the information regarding the neurological dysfunction had been withheld, the imaging protocol only included a range from the skull base to the thighs with oral contrast. This demonstrated no evidence of recurrent systemic disease. The images however did illustrate intense multifocal segmental FDG uptake by the spinal cord with the SUVmax ranging up to 9.6, best demonstrated on the sagittal Maximum Intensity Projection (MIP) image (Fig. 1a). The axial images (Fig. 1b, f, g) localised the abnormal uptake into the intramedullary compartment, while the coronal (Fig. 1c–e) and sagittal (Fig. 1h–j) images confirmed the multifocal nature of the cord abnormalities. No abnormal intracranial activity was present allowing for the limited range imaging protocol, and no uptake was displayed within the optic nerves.

**Fig. 2:**
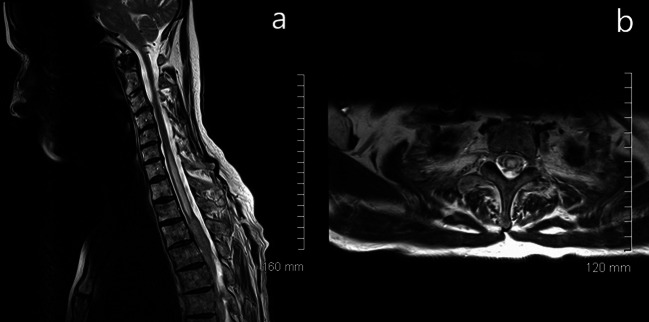
Sagittal T2-weighted MR image (**a**) demonstrates a hyperintense cord lesion extending from C6 to T2/T3. Axial image (**b**) confirms a central location within the cord

Selected sagittal (Fig. 2a) and axial (Fig. 2b) T2-weighted MR images of cervical and upper thoracic spine further supported an intramedullary location of the lesions, differentiating it from the more frequently encountered form of the secondary CNS lymphoma with dominant meningeal spread of the disease.

Following confirmation of the secondary CNS lymphoma by analysis of the cerebrospinal fluid which was positive for high grade B-cell lymphoma both by morphology and flow cytometry, the patient was commenced on a Modified-MATRIX protocol including Methotrexate/Cytarabine chemotherapy intravenously. This resulted in a dramatic halt in the fulminant course of his neurological manifestations, albeit transiently. Due to poor general well-being, repeat ^18^F-FDG-PET/CT was not contemplated but an MR study performed in house suggested partial recovery. He was deemed unfit for an autologous stem cell transplant.

Central nervous system lymphoma is a rare complication of DLBCL and carries a poor prognosis. Primary CNS lymphoma accounts for 0.2–2% of all lymphomas while secondary CNS lymphoma has a higher prevalence of 1–7% (Jellinger and Radiaszkiewicz 1976). The prognosis of primary CNS lymphoma is poor with an overall untreated survival of 1.5 months (Han and Batchelor 2017). The risk of developing secondary CNS disease in a patient with aggressive systemic non-Hodgkin lymphoma is 2–27% and the median survival is 2.2 months (Wang et al. 2014).

Isolated relapse within the CNS is particularly rare with retrospective cohorts numbering just 10–30 patients reported in the literature (Kawano et al. 2012; Patrij et al. 2011; Kim et al. 2010; Stuplich et al. 2012). Furthermore, the pattern of involvement in secondary lymphoma is more commonly leptomeningeal than parenchymal (Hill and Owen 2006; Fonti et al. 2016).

MRI is the first-choice investigation for suspected CNS lymphoma but there is at least one case report of lesions which were detected initially on ^18^FDG-PET/CT with no correlate on MRI (Appaduray et al. 2020).

Risk factors for secondary CNS lymphoma include the involvement of more than one extra-nodal site, advanced stage systemic disease, an elevated international prognostic index and a serum lactate dehydrogenase level greater than three times the upper limit of normal(DeRosa et al. 2014). The risk of secondary CNS disease also depends on the histologic grade of the primary lymphoma. While most secondary CNS lymphomas present with leptomeningeal disease, diffuse large B cell lymphoma most often presents with parenchymal disease (Feugier et al. 2004). The risk of CNS recurrence of an indolent lymphoma is low but, when it does occur, it is usually after histologic transformation to a more aggressive variant (Spectre et al. 2005).

There are several proposed mechanisms for secondary spine involvement which include direct invasion (Costigan and Winkelman 1985), venous spread through Batson’s plexus (Batson 1940), lymphatic dissemination and drop metastases.

The high intensity of uptake seen in our patient on ^18^FDG-PET/CT is pathognomonic of non-Hodgkin lymphoma (Mapelli et al. 2013). Spinal cord lymphoma most commonly presents as a solitary lesion but multifocal lesions are seen in immunocompromised patients (Carnevale and Rubenstein 2016).

Laboratory tests which are helpful to confirm the diagnosis include the cell count, glucose and protein levels, as well as flow cytometry of the CSF and the peripheral blood (D’Cruz et al. 2020). Steroids can create artefacts in CSF cytology and therefore, when possible, lumbar puncture should be performed before commencing steroid treatment.

This case presents a rare example of an isolated lymphoma relapse within the spinal cord. Even more unusual is the multifocal presentation and intramedullary location of the lesions, the latter in contrast to more commonly encountered leptomeningeal involvement. The case highlights the value of ^18^F-FDG-PET/CT in the diagnosis of secondary CNS involvement in patients with treated non-Hodgkin lymphoma which may not always be apparent on MRI. ^18^FDG-PET/CT might delineate the abnormalities more conspicuously and add to the clinical certainty. In addition, more lesions may be identified by the ^18^FDG-PET/CT, which may have an impact on prognosis and support the clinicians in making decisions. When lesions are identified on MRI, as in our case, the intramedullary location is confirmed.

The entire brain should have ideally been included in the imaging range and the importance of conveying an accurate and contemporary clinical history cannot be overemphasised. We speculated that the steroids might have had an impact on the imaging findings and could have conceivably concealed some of the disease activity, such as uptake by the optic nerves. Also, divergent response to the steroid treatment at the cord and optic nerve levels could be a sign of more resistant variant of the disease in the former location. Finally, repeat ^18^F-FDG-PET/CT following dedicated treatment toward the CNS disease would have added further value to the understanding of the disease, its behaviour and nature as well as extent of metabolic response to the treatment. This however was not feasible due to poor general clinical condition of the patient which precluded transfer for a PET/CT.

## Data Availability

Not applicable.
